# Interactions between vitamin D binding protein variants and major dietary patterns on the odds of metabolic syndrome and its components in apparently healthy adults

**DOI:** 10.1186/s13098-019-0422-1

**Published:** 2019-04-08

**Authors:** Mohammad Hossein Rahimi, Mehdi Mollahosseini, Atieh Mirzababaei, Mir Saeed Yekaninejad, Zhila Maghbooli, Khadijeh Mirzaei

**Affiliations:** 1 Department of Community Nutrition, School of Nutritional Sciences and Dietetics, Tehran University of Medical Sciences (TUMS), P.O. Box: 14155-6117, Tehran, Iran; 2 Osteoporosis Research Center, Endocrinology and Metabolism Clinical Sciences Institute, Tehran University of Medical Sciences, Tehran, Iran; 3 Department of Nutrition, School of Public Health, Shahid Sadoughi University of Medical Sciences, Yazd, Iran; 4 Nutrition and Food Security Research Center, Shahid Sadoughi University of Medical Sciences, Yazd, Iran; 5 Department of Epidemiology and Biostatistics, School of Public Health, Tehran University of Medical Sciences, Tehran, Iran

**Keywords:** Dietary patterns, Vitamin D binding protein, Polymorphism, *DBP* gene, *GC* gene, Gc protein, Interaction, Metabolic syndrome

## Abstract

**Background:**

Recent studies have shown that the risks of chronic diseases resulting from high-risk alleles, such as cardiovascular diseases and metabolic syndrome (MetS), can be affected by various dietary patterns. Among the genes affected by environmental factors are those associated with vitamin D binding protein (DBP).

**Methods:**

This cross-sectional study was conducted on a random sample of 265 apparently healthy adults aged 18–50. MetS was defined according to the adult treatment panel III criteria. Major dietary patterns were determined using factor analysis on 24 food groups, using a valid and reliable 147-item food frequency questionnaire (FFQ). *DBP* genotypes were determined by polymerase chain reactions–restriction fragment length polymorphism (PCR–RFLP).

**Results:**

After adjustment for confounder factors, results demonstrated strong interactions between, on the one hand, a high intake of healthy pattern and *DBP* haplotype (rs7041/rs4588 major alleles) and on the other, low MetS odds (OR = 0.64, 95% CI 0.47–0.87, P ≤ 0.001), serum triglyceride levels (OR = 0.72, 95% CI 0.56–0.93, P = 0.01) and fasting blood glucose (OR = 0.36, 95% CI 0.14–0.96, P = 0.04). Also, individuals with a higher adherence to traditional dietary patterns demonstrated reduced odds of high waist circumference among the major allele (low-risk allele) carriers of rs7041/rs4588 (OR = 0.69, 95% CI 0.55–0.88, P = 0. 003). Interactions were also seen between high traditional pattern intake and *DBP* haplotype elevated blood pressure odds (OR = 1.31, 95% CI 1.02–1.68, P = 0.02).

**Conclusions:**

The present evidence indicates that interactions between healthy dietary patterns with *DBP* haplotypes (*Gc 1F*, *Gc 1S* and *Gc 2*) and traditional dietary patterns with DBP haplotypes may be effective in reducing the odds of MetS and some of its components through consuming healthy food groups and inherited low risk alleles.

## Introduction

Metabolic syndrome (MetS) indicates a cluster of cardiovascular disease (CVDs) risk factors associated with high mortality rate, including abdominal obesity, dyslipidemia, hypertension, and insulin resistance [1]. People with MetS have a five-times higher risk of type 2 diabetes (T2D), and are two to three times more at risk of CVDs than those without MetS [2]. In today’s advanced, industrial societies, MetS is an important problem in the development of chronic diseases, thus understanding the factors affecting MetS is a key research challenge [3].

Although food consumption has been correlated with MetS components [4, 5], the role of diet in the development of MetS is not well understood. The application of dietary patterns has recently attracted interest in epidemiological nutrition [6, 7]. In addition, dietary patterns have demonstrated significant relationships with certain plasma risk factors for MetS and metabolic dysfunction [8, 9], such as glycemic indices, plasma lipids and liver enzymes. There are many indications which show that exposure to environmental factors such as dietary patterns, physical activity, and certain genetic traits have effects in the development of this syndrome [10, 11]. Findings from previous studies have shown that exposure to different environmental factors may alter the effects of genetic factors. The risk of chronic diseases resulting from high-risk alleles is also affected by various dietary patterns. Among the genes that are affected by environmental factors are genes associated with vitamin D binding protein (DBP) [10].

DBP, originally known as Gc-globulin (Group-specific component) is the transporter of circulating vitamin D and its metabolites, as well as being active in fatty acid binding and actin scavenging [12]. Proteomic analysis indicates that Gc is a hepatic acute phase reactant and is down-regulated in patients with type I diabetes [13], hepatocellular carcinoma [12, 14], primary non-metastatic breast cancer [15], and sepsis [16]. It is up-regulated in patients with diabetes type II [17], early-stage breast cancer [18], Alzheimer and Parkinson disease [19].

A remarkable *DBP* polymorphism in humans and primates has been demonstrated using various electrophoresis methods [20]. In addition to the three known alleles (*GC1F*, *GC1S* and *GC 2*), over 120 rare variants have been identified, making the Gc-locus amongst the most polymorphic recognized. The initial structure of three major allele-level polymorphisms of *DBP* are according to the combination of two single nucleotide polymorphisms (SNP) located on chromosome 4q12-q13, rs7041 (p. Glutamate 416 Aspartate) and rs4588 (p. Threonine 420 Lysine). These SNPs correspond to allele-level polymorphisms as follows: rs7041 (p. 416 = Asp) + rs4588 (p. 420 = Thr) to *GC*-*1F*, rs7041 (p. 416 = Glu) + rs4588 (p. 420 = Thr) to *GC*-*1S* and rs7041 (p. 416 = Asp) + rs4588 (p. 420 = Lys) to GC-2. The combination of rs7041 (p.416 = Glu) + rs4588 (p.420 = Lys) does not correspond to any of the three major alleles [10]. Of the *GC1* genotypes, *GC1S* is most abundant in European populations, whereas *GC1F* is most abundant among those of African ancestry, with Asians exhibiting intermediate frequencies of both *GC1* forms. The *GC2* form is exceedingly rare in black ethnic groups and is found at similar frequencies in people of Asian and European ancestry [21].

Concentrations of 25-hydroxyvitamin D (25(OH)D) are related to DBP concentration, whereas any type of DBP phenotype has its own specific amount of 25(OH)-vitamin D, with the hierarchy of affinity being *GC1F*>*GC1S*>*GC2*. In addition, this hierarchy (*GC1F*>*GC1S*>*GC2*) also applies to the relative abundance of the respective forms in human serum [22].

There is much evidence about the associations between vitamin D metabolism, lipolysis and MetS, and some studies have shown the association of MetS with changes in vitamin D concentrations [23–25]. However, it is not known whether the DBP polymorphism affects the metabolic syndrome and its components through changes in vitamin D concentrations.

Although several environmental and genetic factors have been established in relation to metabolic dysfunction [26, 27], few studies have investigated the interactions of dietary patterns and genetic variants with MetS; most of them have focused primarily on nutrients [28]. In this study, the interactions between dietary patterns and *DBP* polymorphism rs7041/rs4588 separately and combined (*GC1F*, *GC1S* and *GC 2* alleles) were investigated in terms of the odds of MetS and its components.

## Methods and materials

### Study population

The study population was recruited using advertising from July 2015 to June 2016 across the West and Central regions of Tehran according to cluster sampling techniques, and using community-based sampling. 363 participants (18–50 years) were enrolled, 98 participants were excluded and 265 participants who were apparently healthy and not known to have MetS, took part in this cross-sectional study and were included with the following criteria: aged 18–50 years, no smoking and alcohol use, absence of any acute or chronic inflammatory disease, and not being pregnant during the study. Participants with a history of CVDs, T2D, cancer or stroke and dietary supplement use (including vitamin D), were excluded because of possible disease-related changes in diet, as well as those taking any therapeutic medications. Also, all participants who did not respond to more than 70 food items from the FFQ, or whose energy consumption lay outside the range 800 kcal/day (3347 kJ/day) to 4200 kcal/day (17,573 kJ/day), [29] were not included in the study.

### Measurement of biochemical parameters

After completing the consent letter, the blood samples were collected into tubes containing 0.1% EDTA after 10–12 h fasting, then immediately centrifuged, aliquoted and stored at a temperature of − 80 °C. All samples were evaluated by the same method and under similar conditions. Serum 25(OH)D3 was measured using a Biosource kit (Bio-source Europe S.A, Belgium) by the radioimmunoassay (RIA) method. Serum glucose level was measured by the glucose oxidase phenol 4-aminoantipyrine peroxidase (GOD/PAP) method, triglycerides (TG) with the glycerol-3-phosphate oxidase phenol 4-aminoantipyrine peroxidase (GPO-PAP) method, total cholesterol by endpoint enzymatic method, and high-density lipoprotein cholesterol (HDL-C) with an enzymatic clearance assay. All of the above measurements were made using Randox laboratory kits (Hitachi 902 Analyzer; Hitachi LTD, Tokyo, Japan). Quality control was carried out for all biochemical assessments using the control serum, as in the manufacturer’s instructions.

### Metabolic syndrome criteria

MetS was defined according to the adult treatment panel III (ATPIII) criteria [30]. A subject has MetS when at least 3 of the fol-lowing 5 risk factors are present: (1) hyperglycemia as FBS ≥ 100 mg/dl (5.6 mmol/l); (2) hypertriglyceridemia as serum TG ≥ 150 mg/dl (1.69 mmol/l); (3) low HDL-C serum < 40 mg/dl (1.04 mmol/l) for men, and < 50 mg/dl (1.29 mmol/l) for women; (4) hypertension as BP ≥ 130/85 mmHg; and (5) abdominal adiposity (defined as waist circumference 88 cm [women] or 102 cm [men]).

### Assessment of anthropometric measures

Height was measured in a standing position without shoes, using a tape meter, while the shoulders were in a normal state (with a precision of 0.5 cm). Weight was measured while the subjects were minimally clothed without shoes, using digital scales (with an accuracy of 0.1 kg). WC was measured at the narrowest point, and hip circumference at the widest point, over light clothing, using a non-elastic tape meter, without any pressure to the body surface [31].

### Assessment of blood pressure

After the participants sat in a relaxed position for 15 min, blood pressure was measured using a standardized sphygmomanometer [32].

### Assessment of other variables

Additional covariate information regarding physical activity, smoking habits, medical history, and current use of medication was obtained using questionnaires completed during the screening. The validated International Physical Activity Questionnaire (IPAQ) was used to obtain data on physical activity [33]. This questionnaire has also been validated in Iran [34]. It assesses physical activity across a complete set of domains, including domestic, leisure time and gardening activities, as well as work-related and transport-related activities. Data were obtained on the frequency and time spent on light, moderate and hard intensity activities, according to the list of common daily activities over the past year [35].

### Dietary intake assessment and extraction of dietary patterns

Information about food intake was collected with a semi-quantitative food frequency questionnaire (FFQ). The FFQ, originally developed for this study, was a Tehran Lipid and Glucose Study (TLGS) format questionnaire, and contained questions about the average consumption and consumption frequency of 147 food items during the past year [36]. The food items were chosen according to the most frequently consumed items in the national food consumption survey in Iran [37]. Subjects illustrated their food consumption frequencies on a daily basis (bread), weekly basis (rice and meat), monthly basis (fish), yearly basis (organ meats), or a never/seldom basis according to portion sizes provided in the FFQ. The FFQ was based on food items rather than dishes, since different recipes are used in food preparation (for example, beans, different meats and oils, and rice). In order to complete the FFQ, the interview session took about 45 min. The interviewer read out the food items on the FFQ, and recorded their serving size and frequency. The weight of seasonal fruits was estimated according to the number of seasons within which each food was available. Daily intakes of each food item were determined based on the portion size or household measures, and multiplied by the consumption frequency of each food item [38].

Based on the similarity of nutrients, food items were grouped into 24 pre-defined food groups. This classification was based on the similarity of their nutrients, according to previous studies [39]. If the nutrients composition of a food item was considerably different from other items (like eggs, margarine, tea and coffee), or if its consumption was a special eating habit (like butter), it was considered as an independent group. Then, the adjusted means for energy were calculated for each food group using the residual method [40]. In this way, the adjusted energy values were obtained.

In the next step, to determine the suitability of the model, the Kaiser–Meyer–Olkin (KMO) and the Bartlett test were used. Dietary patterns were determined by factor analysis (FA). The principal component analysis method (PCA) with varimax rotation was applied to these energy-adjusted food groups. The factors obtained were checked on the basis of the Eigen-values of energy-adjusted food groups; each factor having an Eigen-value of greater than 1.5 was considered as a major dietary pattern. The designation of patterns was based on the interpretation of food items in each factor, which together accounted for 27.9% of the total variation, on the basis of the scree plot and varimax rotation in the 24 food groups [41]. It should be noted that other food patterns were identified, but were not considered due to their low variance. Then, the subjects were categorized into quartiles, according to dietary patterns. The major dietary patterns were identified as follows, on the basis of previous knowledge:

A. Healthy dietary pattern: rich in green vegetables, liquid oils and olive oil, fish and poultry, fruits, legumes and eggs, but low in high-energy drinks, sweets and snacks.
B. Traditional dietary pattern: high-fat dairy products, dry fruits, starchy vegetables, seeds, legumes, and seasonings were the main components. The main feature of this dietary pattern was fiber consumption.
C. Western dietary patterns: high consumption of refined grains, high-energy drinks and beverages, solid fats, mayonnaise, low-fat dairy products; limited consumption of unrefined grains, vegetables, liquid oils and low-fat dairy products.

### DNA extraction

Genomic DNA was extracted from whole blood using the Mini Columns Type kit. The Gene ALL DNA kit (Gene ALL, Korean) was used according to the manufacturer’s protocol. The concentration and purity of extracted DNA was measured using a Nano Drop ND-1000 spectrophotometer. DNA was stored at − 20 °C until ready for use. The *DBP* polymorphisms in exon 11 [Asp416Glu and Thr420Lys] were determined by polymerase chain reactions–restriction fragment length polymorphism (PCR–RFLP). The PCR procedures were carried out in a total volume of 30 µl with 50 ng of genomic DNA, 2.5 U Blue Master Mix-Tembase Buffer, 1 mmol Dimethyl sulfoxide, 0.2 mM of each dNTP, and 10 pmol of each primer (forward: 5′-CAAGTCTTATCACCATCCTG-3′ and reverse: 5′-GC CAAGTTACAATAACAC-3′) [42]. PCR amplifications were performed according to the following cycling conditions: initial denaturation at 94 °C for 5 min, followed by 35 cycles at 94 °C for 45 s, 60 °C for 1 min, and 72 °C for 1 min, with a final extension at 72 °C for 10 min.

Then, while being kept at 37 °C overnight, 10 µl of the amplified products were digested in a 20 µl reaction containing either 2.5 U *Hae*III or 2.5 U *Sty*I restriction enzymes (Fermentas, Germany) for both Asp, 416, Glu and Thr, 420, Lys polymorphisms, respectively. The digestion products were visualized, stained with ethidium bromide on 2% agarose gel. Individuals homozygous for the Glu allele showed two bands (577 and 232 bp) in the DBP Asp, 416, Glu polymorphism while individuals homozygous for the Asp allele had a nondigested band at 809 bp. The homozygous Thr allele of the Thr, 420, Lys polymorphism appeared as a single band (809 bp), while the Lys allele showed two bands (584 and 225 bp). *DBP* haplotypes were determined by observing the digestion products of both restriction enzymes. The *GC1S* had the *Hae*III but not the *Sty*I site. The *GC1F* had neither the *Hae*III nor the *Sty*I site. The *GC2* had the *Sty*I but not the *Hae*III site. The existence of both restriction sites on a single haplotype has not yet been described [43]. We checked DNA purity by nanodrop and to confirm the PCR-RFLP results, 10% of the PCR samples were directly sequenced.

The minor allele (risk allele) frequency for rs7041T was 0.488, while that of rs4588A was 0.235. They were divided into six main genotypes, which included three homozygotes, *GC1F*-*1F, GC1S*-*1S* and *GC2*-*2*, and heterozygotes, *GC1F*-*1S*, *GC2*-*1F* and *GC2*-*1S*. The genotypes were coded as follows: code 0 for (*GC1F/F*), code 1 for (*GC1F/S*), code 2 for (*GC1F/2*), code 3 for (*GC1S/S*), code 4 for (*GC1S/2*) and code 5 for (*GC2/2*). The Haplotypes were coded as follows: code 0 for (*GC1F*), code 1 for (*GC1S*) and code 2 for (*GC2*), based on the risk allele carrier (Table 1).

**Table 1:** Allele and genotype frequencies of rs7041 and rs4588 polymorphisms among subjects

rs7041		rs4588		Haplotypes of rs7041 and rs4588	
Alleles (n = 526)					
Allele	Frequency	Allele	Frequency	Alleles	Frequency
G	51.12	C	76.42	GC	44.2
				GA	6.8
T	48.88	A	23.57	TC	32.1
				TA	16.7

Genotypes (N = 263)					
Genotype	Frequency	Genotype	Frequency	Haplotype	Frequency
GG	26.4	CC	52.8	TT CC	4.9
				TG CC	21.1
GT	48.7	CA	46	TT CA	19.9
				GG CC	26.4
TT	24.2	AA	0.4	TG CA	27.5
				TT AA	0.4

Haplotype name	Combination of genotypes of rs7041, rs4588 (N)				
Haplotype (GC1F/F)	TT CC (13)				
Haplotype (GC1F/S)	TG CC (57)				
Haplotype (GC1F/2)	TT CA (50)				
Haplotype (GC1S/S)	GG CC (70)				
Haplotype (GC1S/2)	TG CA (72)				
Haplotype (GC2/2)	TT AA (1)				
Total	263				
Haplotype 1 (GC1F)	T-C (133)				
Haplotype 2 (GC1S)	G-C (269)				
Haplotype 3 (GC2)	T-A (124)				
Total	526				

*N* number of study subjects, *n* number of alleles

### Statistical analysis

The normality of distribution was tested by applying Kolmogorov–Smirnov’s test. Data on quantitative characteristics were reported as the mean ± SD, and data on qualitative characteristics were expressed as a percentage. Qualitative variables were compared using an analysis of variance (ANOVA), and an independent t-test was used to compare the quantitative variables.

To investigate the interactions between dietary patterns and haplotypes of rs7041 and rs4588, binary logistic regression models were used on the qualitative variables of MetS and its components. Moreover, age, sex, physical activity (PA) and vitamin D 25(OH)D3 concentrations were considered as confounders, and adjusted analysis was performed in these models. The level of significance was set at a probability of ≤ 0.05 for all tests, and P < 0.1 was considered as marginal significance. Statistical analysis was performed using SPSS version 22.0 (SPSS, Chicago, IL, USA).

## Results

### Descriptive data

This comparative cross-sectional study was conducted on 265 participants (52.5% female). The mean ± SD of age, height, BMI, and weight of individuals with MetS were 39.45 ± 7.8 (years), 168.57 ± 10.7 (cm), 31.05 ± 4.3 (kg/m^2^), and 88.65 ± 16.9 (kg), respectively. It was found that 12.5% of participants had MetS, comprising 21 males and 12 females. The P-values for all individual variables, with and without MetS, were determined with the ANCOVA model, after adjusting for age, sex, weight and PA. The results revealed that individuals with MetS had statistically lower means of serum HDL-C levels (P ≤ 0.0001), and higher means of weight (P ≤ 0.0001), BMI (P ≤ 0.0001), WC (P = 0.01), systolic blood pressure (P = 0.02), diastolic blood pressure (P ≤ 0.0001) and serum TG levels (P ≤ 0.0001) (Table 2).

**Table 2:** Study population characteristics with MetS and without MetS

	With MetS (n = 33)		Without MetS (n = 232)		P value*
	Mean	SD	Mean	SD	
Demography					
Age (years)	39.45	7.8	34.46	8.7	< *0.0001*
Weight (kg)	88.65	16.9	71.31	14.21	< *0.0001*
Height (cm)	168.57	10.7	168.18	9	0.57
BMI (kg/m^2^)	31.05	4.3	25.17	4.5	< *0.0001*
WC (cm)	102.02	9.6	86.78	11.63	*0.01*
Blood pressure					
Systolic BP (mmHg)	12.61	1.2	11.84	1.25	*0.02*
Diastolic BP (mmHg)	8.34	1.1	7.64	0.8	< *0.0001*
Blood parameters					
FBS (mg/dl)	96.21	15.9	93.33	17.5	0.46
TG (mg/dl)	237.03	125.7	110.18	79.5	< *0.0001*
Total-Chol (mg/dl)	193.45	22.0	183.32	42.1	0.55
HDL-C (mg/dl)	38.33	6.7	50.27	11.4	< *0.0001*
LDL-C (mg/dl)	106.48	14.42	100.54	28.5	0.96

Italic values indicate a significant difference (P ≤ 0.05)

*BMI* body mass index, *WC* waist circumference, *BP* blood pressure, *HDL-C* HDL-cholesterol, *LDL* low density lipoprotein, *FBS* fasting blood sugar, *TG* triglyceride, *MetS* metabolic syndrome, *SD* standard deviation, *Total-Chol* total cholesterol

* After adjustment for age, sex, weight and physical activity

### Characteristics of major dietary patterns extracted

The factor-loading matrices for three dietary patterns are shown in Table 3. Some food groups have a positive factor load, and some have a negative factor load. A positive factor load of the food groups in a dietary pattern shows a positive relationship, and a negative factor load shows a negative relationship between the food group and the dietary patterns. The greater the factor load of a food group in a given dietary pattern, the higher the proportion it plays within that food group. The healthy dietary pattern, the traditional dietary pattern, and the western dietary pattern explained 8.79%, 7.98% and 11.15% of the total variance, respectively.

**Table 3:** Factor loadings for the three identified dietary patterns among the study participants

Food groups	Dietary patterns		
	Western	Traditional	Healthy
Refined grain	0.664	–	–
Unrefined grain	− 0.652	0.304	–
Liquid oil	− 0.598	–	
Vegetable	− 0.593	–	0.307
Low-fat dairy	− 0.518	–	
High-energy drinks and beverages	0.387	–	− 0.344
Solid fat	0.372	–	–
Mayonnaise	0.314	–	–
Processed foods	–	–	–
Butter	–	–	–
Seasonings	–	0.673	–
Fruits	–	0.511	–
Seeds	–	0.457	–
Starchy vegetables	–	0.452	–
High-fat dairy	–	0.386	–
Dry fruits	–	0.324	
Sweets	–	–	− 0.635
Snacks	–	–	− 0.580
Legumes	–	0.391	0.565
Egg	–	–	0.331
Fish and poultry meat	–	–	0.307
Organ meats	–	–	–
Red meat	–	–	–
Tea and coffee	–	–	–
Total variance (%)	11.15	8.79	7.98

### DBP gene-dietary pattern interactions for MetS components

When possible interactions between dietary patterns and the haplotypes were tested on serum TG levels, strong gene-diet interactions were found between the haplotypes and the healthy pattern, in terms of reduced levels of serum TG, in the regression model. Interactions between the healthy pattern and haplotypes on reduced levels of serum TG were significant after adjusting for confounders in the adjusted model (OR = 0.72, 95% CI 0.56–0.93, P for interaction = 0.01), while being non-significant before adjustment.

A similar interaction was observed between the healthy pattern and haplotypes in terms of reduced FBS levels, which were significant after adjusting for confounders in the regression model (OR = 0.36, 95% CI 0.14–0.96, P for interaction = 0.04).

In addition, an interaction between the healthy pattern and haplotypes in terms of reduced blood pressure was marginally significant, before and after adjusting for confounders, in two models of the regression model (OR = 0.79, 95% CI 0.62, 1.01, P for interaction after adjustment = 0.06). However, the interaction between the traditional pattern and haplotypes showed significant increases in blood pressure after adjusting for confounders (OR = 1.31, 95% CI 1.02–1.68, P for interaction = 0.02).

Also, the interaction between the traditional pattern and haplotypes was significant in terms of reduced WC, after adjusting for confounders, in two models of the regression model (OR = 0.69, 95% CI 0.55–0.88, P for interaction = 0.003) (Table 4).

**Table 4:** Investigation of the interactions between major dietary patterns and GC1F, GC1S and GC2 haplotypes^c^ on the risk of MetS

MetS components	Dietary pattern^a^		
	Healthy	Traditional	Western
TG			
Crude model			
P-interaction	0.20	0.69	0.51
OR (%95CI)	0. 88 (0.73, 1.06)	1. 03 (0.86, 1.25)	1. 06 (0.87, 1.29)
Adjusted model^b^			
P-interaction	*0.01*	0.33	0.79
OR (%95CI)	*0. 72 (0.56, 0.93)*	1.13 (0.71, 0.99)	1. 03 (0.78, 1.36)
HDL			
Crude model			
P-interaction	0.36	0.74	0.65
OR (%95CI)	0. 92 (0.79, 1.09)	1.02 (0.87, 1.19)	1. 03 (0.87, 1.22)
Adjusted model			
P-interaction	0.60	0.18	0.87
OR (%95CI)	0. 95 (0.78, 1.15)	1. 14 (0.93, 1.39)	1. 01 (0.82, 1.26)
WC			
Crude model			
P-interaction	0.99	*0.04*	0.01
OR (%95CI)	1. 00 (0.84, 1.78)	*0. 84 (0.71, 0.99)*	1. 25 (1.04, 1.21)
Adjusted model			
P-interaction	0.99	*0.003*	0.70
OR (%95CI)	0. 99 (0.80, 1.24)	*0. 69 (0.55, 0.88)*	1. 04 (0.81, 1.34)
FBS			
Crude model			
P-interaction	0.12	0.59	0.21
OR (%95CI)	1. 39 (0.91, 2.12)	0. 88 (0.55, 1.40)	0. 74 (0.46, 1.18)
Adjusted model			
P-interaction	*0.04*	0.11	0.11
OR (%95CI)	*0. 36 (0.14, 0.96)*	0. 51 (0.22, 1.18)	0. 46 (0.18, 1.21)
BP			
Crude model			
P-interaction	*0.09*	0.16	0.95
OR (%95CI)	*0. 85 (0.70, 1.02)*	1. 14 (0.94, 1.37)	0. 99 (0.81, 1.20)
Adjusted model			
P-interaction	*0.06*	*0.02*	0.64
OR (%95CI)	*0. 79 (0.62, 1.01)*	*1. 31 (1.02, 1.68)*	0. 94 (0.72, 1.22)
MetS			
Crude model			
P-interaction	0.12	0.91	0.16
OR (%95CI)	0. 83 (0.85, 1.05)	0. 98 (0.78, 1.24)	1. 19 (0.93, 1.52)
Adjusted model			
P-interaction	< *0.001*	0.80	0.44
OR (%95CI)	*0. 64 (0.47, 0.87)*	1. 03 (0.77, 1.40)	1. 13 (0.82, 1.57)

Italic values indicate a significant difference (P ≤ 0.05)

*FBS* fasting blood sugar, *TG* triglyceride, *HDL* high density lipoprotein, *WC* waist circumference, *MetS* metabolic syndrome, *BP* blood pressure

^a^ First quartile is used as reference (low adherence)

^b^ Adjusted for sex, age, physical activity and vitamin D

^c^ GC1F is used as reference (low risk)

### DBP gene-dietary pattern interactions for MetS

The healthy pattern interacted with haplotypes in relation to the odds of MetS. Increased adherence to the healthy pattern (compared*GC1S* to the first quartile) was associated with a lower odds of MetS among the major allele carriers of rs7041/rs4588 (*GC1F*, and *GC2* respectively). When vitamin D concentrations were analysed as a confounder, along with sex, age and PA, the differences in the odds of MetS became more obvious (OR = 0.64, 95% CI 0.47–0.87, P for interaction ≤ 001) (Table 4).

In individuals with the major homozygote of rs7041, higher adherence to the healthy and traditional patterns (compared to their first quartiles) were associated with lower odds of MetS (OR = 0.57, 95% CI 0.41–0.79, P for interaction ≤ 0.001 and OR = 0.61, 95% CI 0.43–0.87, P for interaction = 0.006). However, those with higher adherence to the western dietary pattern and minor allele carriers had increased odds of MetS (OR = 1.37, 95% CI 0.97–1.95, P for interaction = 0.060) (Table 5).

**Table 5:** Investigation on interactions between major dietary patterns and rs7041 genotype^a^ on the odds of MetS

	Dietary pattern^b^		
	Healthy dietary pattern	Traditional dietary pattern	Western dietary pattern
Crude model			
P-interaction	*0.01*	0.73	0.36
OR (%95CI)	*0.74 (0.58, 0.93)*	1.04 (0.81, 1.32)	1.11 (0.88, 1.42)
Adjusted model^c^			
P-interaction	< *0.001*	*0.006*	*0.060*
OR (%95CI)	*0.57 (0.41, 0.79)*	*0.61 (0.43, 0.87)*	*1.37 (0.97, 1.95)*

Interaction of dietary patterns and rs7041 genotypes on the qualitative variables of MetS measured using binary logistic regression

Italic values indicate a significant difference (P ≤ 0.05)

*MetS* metabolic syndrome

^a^ T is used as reference (risk allele)

^b^ First quartile is used as reference (low adherence)

^c^ Adjusted for sex, age, physical activity and vitamin D

Interactions between the risk allele of rs4588 and low intake of the traditional pattern were not significant (P for interaction = 0.18), but in individuals with the major homozygote of rs4588, higher adherence to the healthy pattern (compared to the first quartile) was associated with lower odds of MetS (OR = 0.22, 95% CI 0.13–0.36, P for interaction ≤ 0.0001). This stands in contrast to the western dietary pattern and minor allele carriers, who had increased odds of MetS (OR = 1.71, 95% CI 1.07–2.75, P for interaction = 0.02) (Table 6).

**Table 6:** Investigation of the interactions between dietary patterns and rs4588 genotype^a^ on the risk of MetS

	Dietary pattern^b^		
	Healthy dietary pattern	Traditional dietary pattern	Western dietary pattern
Crude model			
P-interaction	< *0.0001*	0.93	*0.01*
OR (%95CI)	*0.50 (0.35, 0.71)*	1.01 (0.73, 1.40)	*1.56 (1.10, 2.20)*
Adjusted model^c^			
P-interaction	< *0.0001*	0.18	*0.02*
OR (%95CI)	*0.22 (0.13, 0.36)*	0.74 (0.47, 1.16)	*1.71 (1.07, 2.75)*

Interaction of dietary patterns and rs4588 genotypes on the qualitative variables of MetS measured using binary logistic regression

Italic values indicate a significant difference (P ≤ 0.05)

*MetS* metabolic syndrome

^a^A is used as reference (risk allele)

^b^First quartile is used as reference (low adherence)

^c^Adjusted for sex, age, physical activity and vitamin D

## Discussion

In the present cross-sectional study of Tehrani adults, the interactions between dietary patterns and genetic variants of *DBP* SNPs (rs7041, rs4588) were assessed in relation to MetS risk levels and MetS components. The two SNPs (rs7041 and rs4588) were found to be in linkage disequilibrium (D′ = 1, r^2^ = 0.23), which meant that there was a nonrandom association between alleles of the two SNPs.

The interactions between all three dietary patterns and rs7041 showed noteworthy effects on the odds of MetS. In two of the regression models, even after adjusting for confounders, the significant interaction between the healthy pattern, rs7041, and MetS did not change. Beyond this, two strong interactions were observed between rs4588 and both the healthy pattern and western pattern. An interaction between the quartiles of the healthy pattern and rs4588 alleles had strongly significant effects on reducing MetS odds, exactly like the interaction effect between the healthy pattern and rs7041. On the contrary, a higher intake of the western pattern among the risk allele carriers of rs4588 was associated with higher odds of MetS. However, significant interactions between traditional and western patterns with rs7041 were seen only after adjusting for the effect of vitamin D. This can be seen as a demonstration of the effects of *DBP* gene variants on the bioavailability of vitamin D (according to past studies [44, 45]), and an indication of vitamin D’s subsequent effects on MetS components. The mechanisms of this relationship are not clear. But, 1,25(OH)_2_ vitamin D3 is essential for normal insulin secretion and there is a possibility that DBP is a carrier protein for vitamin D and the affinity of DBP for 25-OH-vitamin D3 and 1,25 (OH)_2_ vitamin D3 varies depending on the genotype of DBP [46]. DBP genotype have been previously shown to be strongly associated with circulating 25-OH-vitamin D3 in diverse populations [47, 48] and in genome-wide association studies [49, 50].

In addition, subsequent to Baier et al.’s [51] initial report describing the linkage of this locus to prediabetic phenotypes in Pima Indians, the Gc locus has been reported to be associated with noninsulin-dependent diabetes mellitus in Japan [52].

In the ARIC study [53], lower compared with higher serum concentrations of 25(OH)D were associated with major incidents of strokes, over nearly 20 years of follow-up. This association was similar by race, but there was suggestive evidence (though not statistically significant), that associations between low 25(OH)D and stroke incidence were stronger among participants genetically disposed to higher serum DBP (i.e. those with presence of rs7041 G allele and rs4588 A allele). Individuals with a rs7041G allele (versus a T allele) and individuals with a rs4588 A allele (versus a C allele) have been shown to have higher DBP levels, and therefore lower levels of bioavailable 25(OH)D for a given 25(OH)D level. In this study, people who had a higher adherence to the healthy pattern and had rs7041 G allele and rs4588 A allele were less likely to be at risk of MetS, and vice versa.

According to the authors’ knowledge, this study is the first study to examine the interactions between dietary patterns and *DBP* gene variants on MetS. However, among studies that examine the interactions between dietary patterns and other genes on MetS, the study of Flips et al. [54] should be mentioned; it showed that an interaction between dietary fat, especially PUFA, and ACSL1 gene polymorphisms, is effective in reducing the risk of MetS. Therefore, it can be concluded that—to a large extent—the success of nutritional interventions for controlling weight, diabetes, and MetS components depends on the genetics of different individuals.

A noteworthy point about the traditional dietary pattern in this study is its high fiber content, which according to past studies [55], especially Whelton et al.’s meta-analysis [56], seems to lead to reduced systolic blood pressure. Traditional food patterns identified in the present study were not similar to patterns extracted in other studies [57], in that high-fat dairy products, starchy vegetables and legumes were similar components in this study and other studies, while red meat and butter were not. Furthermore, the consumption of healthy food items was observed in this pattern, including fruits and unrefined grains. Thus, by taking these items into consideration, this pattern could also be termed a fiber-rich pattern, or a neo-traditional pattern; its interaction with DBP leads to reductions in WC and blood pressure.

The protective effect of healthy patterns in interacting with the *DBP* gene on FBS levels is also due to its low consumption of sweets, as well as high-energy drinks and beverages. Also, the effect of the healthy pattern on serum TG levels and MetS, in addition to the frequent consumption of items like vegetables and fish, can be seen in milk consumption. Low-fat dairy products score highly in the healthy pattern, and milk appears to be insulinotropic [58].

In addition to the effect of the patterns, there are at least two reasons why some associations between the DBP haplotypes and MetS components can be suspected. First, the metabolically active form of vitamin D is involved in a feedback system of insulin regulation, because DBP binds vitamin D [59]. Second, DBP plasma levels are under genetic control: almost 84% of the variation in DBP protein is explained by genes, and the remainder by environmental factors, some of which may be sex-specific [60]. *GC* variants have formerly been associated with type 2 diabetes in seven Polynesian populations, and insulin regulation and glucose in a Hispanic-American/Anglo population of Southern Colorado and Dogrib Indians of Canada [59]. In this study, blood pressure was measured only once, which could suggest low precision in the hypertension estimate, causing attenuated relationships. However, with these conditions, the significant interaction of the traditional dietary pattern and the *DBP* gene was only observed with systolic blood pressure in the regression model before adjustment for vitamin D concentrations.

To the authors’ knowledge, one strength of the current study is that it is the first study to evaluate the interactions between *GC* gene variants and major dietary patterns on the odds of MetS and its components. Additionally, because it included only Caucasians with Iranian ancestry, its homogeneous population was a strength (and therefore results were less likely to be affected by population stratification). However, in the interpretation of this study, certain limitations should be taken into consideration. First, the main limitation of the present study was its low sample size, which probably led to insufficient sample for the statistical analyses for participants presented with the metabolic syndrome. Second, dietary patterns can vary by socioeconomic status, ethnic group, and culture. Thus, it is necessary to replicate the results of the current study in other populations. Also, the genetic variants identified only account for a very small section of the variability in vitamin D status, and this study is consistent with other studies based on protein isoforms of this binding protein [61, 62]. Also, there is consistent support for one SNP in the enzyme that converts the storage form of the vitamin (25(OH)D) to the active ligand 1-25(OH)D (CYP27B1; rs10877012) [63], and one SNP in the vitamin D receptor gene (*VDR*; rs10735810) [64]. As mentioned above, genetic variants, through changes in vitamin D status, cause changes in health outcomes, including in the components of metabolic syndrome.

While the body of evidence is still incomplete, a more comprehensive assessment of gene-diet interactions with large sample size to have enough power, will need to include more genetic risk factors for MetS when they are identified, and to perform analyses on the interactions between specific variants and dietary intakes.

## Conclusion

The reality is that MetS is widespread in western societies, and its incidence has clearly also increased in developing countries which have shifted to a westernized lifestyle. This highlights the critical role played by a westernized diet and lifestyle in MetS incidence [65]. The present evidence indicates that interactions between healthy and traditional dietary patterns with DBP haplotypes (*GC1F*, *GC1S* and *GC2*) may be effective in reducing the odds of MetS and its components. Moreover, the current study’s data suggest that the effects of a western dietary pattern on MetS odds are not homogeneous in people of various genetic backgrounds. High intakes of the western pattern increased the odds of MetS among those with a greater genetic susceptibility to this disease. The data also show that refined grains and high-energy drinks and beverages may be the main foods driving the interactions between a western dietary pattern and genetic variations in determining MetS odds. High intakes of these foods significantly increased the risk of MetS among individuals carrying more risk alleles of the *DBP* variants.

## Abbreviations

ATPIII: adult treatment panel III
BMI: body mass index
25(OH)D: 25(OH)-vitamin D
CVDs: cardiovascular diseases
DNA: deoxyribonucleic acid
FFQ: food frequency questionnaire
FA: factor analysis
Glu: glutamate
Asp: aspartate
Thr: threonine
Lys: lysine
FBS: fasting blood sugar
KMO: Kaiser–Meyer–Olkin
LDL: low-density lipoprotein
MetS: metabolic syndrome
PA: physical activity
PCR–RFLP: polymerase chain reactions–restriction fragment length polymorphism
SNP: single-nucleotide polymorphism
T2D: type 2 diabetes
TG: triglycerides
WC: waist circumference
OR: odds ratio

## References

[1] Grundy SM, American Heart Association, National Heart, Lung and Blood Institute (2005). Diagnosis and management of the metabolic syndrome: an American heart association/national heart, and blood institute scientific statement. Circulation.

[2] Grundy SM (2007). Metabolic syndrome: a multiplex cardiovascular risk factor. J Clin Endocrinol Metab.

[3] Alberti KGMM, Zimmet P, Shaw J (2006). Metabolic syndrome—a new world-wide definition. A consensus statement from the international diabetes federation. Diabet Med.

[4] Coates AM, Howe PR (2007). Edible nuts and metabolic health. Curr Opin Lipidol.

[5] Van Dam RM, Hu FB (2005). Coffee consumption and risk of type 2 diabetes: a systematic review. JAMA.

[6] Schulze MB, Hu FB (2002). Dietary patterns and risk of hypertension, type 2 diabetes mellitus, and coronary heart disease. Curr Atheroscler Rep.

[7] Hu FB (2002). Dietary pattern analysis: a new direction in nutritional epidemiology. Curr Opin Lipidol.

[8] Williams DE (2000). A cross-sectional study of dietary patterns with glucose intolerance and other features of the metabolic syndrome. Br J Nutr.

[9] Mollahosseini M (2017). The association between fruit and vegetable intake and liver enzymes (aspartate and alanine transaminases) in Tehran, Iran. Ethiop J Health Sci.

[10] Malik S (2013). Common variants of the vitamin D binding protein gene and adverse health outcomes. Crit Rev Clin Lab Sci.

[11] Kissebah AH (2000). Quantitative trait loci on chromosomes 3 and 17 influence phenotypes of the metabolic syndrome. Proc Natl Acad Sci.

[12] Gressner OA (2009). Inverse association between serum concentrations of actin-free vitamin D-binding protein and the histopathological extent of fibrogenic liver disease or hepatocellular carcinoma. Eur J Gastroenterol Hepatol.

[13] Blanton D (2011). Reduced serum vitamin D-binding protein levels are associated with type 1 diabetes. Diabetes.

[14] Chan KY (2006). Positional expression profiling indicates candidate genes in deletion hotspots of hepatocellular carcinoma. Mod Pathol.

[15] Kim BK (2009). The multiplex bead array approach to identifying serum biomarkers associated with breast cancer. Breast Cancer Res.

[16] Hattori N (2009). YKL-40 identified by proteomic analysis as a biomarker of sepsis. Shock.

[17] Cho EH (2007). The discovery of biomarkers for type 2 diabetic nephropathy by serum proteome analysis. Proteomics Clin Appl.

[18] Pawlik TM (2006). Proteomic analysis of nipple aspirate fluid from women with early-stage breast cancer using isotope-coded affinity tags and tandem mass spectrometry reveals differential expression of vitamin D binding protein. BMC Cancer.

[19] Zhang J (2008). CSF multianalyte profile distinguishes Alzheimer and Parkinson diseases. Am J Clin Pathol.

[20] Constans J (1987). Polymorphism of the vitamin D binding protein (DBP) among primates: an evolutionary analysis. Am J Phys Anthropol.

[21] Chun RF (2012). New perspectives on the vitamin D binding protein. Cell Biochem Funct.

[22] Lauridsen AL, Vestergaard P, Nexo E (2001). Mean serum concentration of vitamin D-binding protein (Gc globulin) is related to the Gc phenotype in women. Clin Chem.

[23] Minambres I (2015). Hypovitaminosis D in type 2 diabetes: relation with features of the metabolic syndrome and glycemic control. Endocr Res.

[24] Schmitt EB (2018). Vitamin D deficiency is associated with metabolic syndrome in postmenopausal women. Maturitas.

[25] Makariou S (2012). The relationship of vitamin D with non-traditional risk factors for cardiovascular disease in subjects with metabolic syndrome. Arch Med Sci.

[26] Qi L (2009). Genetic predisposition, Western dietary pattern, and the risk of type 2 diabetes in men. Am J Clin Nutr.

[27] Sánchez-Moreno C (2011). APOA5 gene variation interacts with dietary fat intake to modulate obesity and circulating triglycerides in a Mediterranean population. J Nutr.

[28] Song Y, Joung H (2012). A traditional Korean dietary pattern and metabolic syndrome abnormalities. Nutr Metab Cardiovasc Dis.

[29] Willett W, Stampfer M (1998). Implications of total energy intake for epidemiologic analysis. Monogr Epidemiol Biostat.

[30] Grundy SM (2005). Diagnosis and management of the metabolic syndrome: an American Heart Association/National Heart, Lung, And Blood Institute scientific statement. Circulation.

[31] Janssen I, Katzmarzyk PT, Ross R (2002). Body mass index, waist circumference, and health risk: evidence in support of current National Institutes of Health guidelines. Arch Intern Med.

[32] Group S.R. (2015). A randomized trial of intensive versus standard blood-pressure control. N Engl J Med.

[33] Craig CL (2003). International physical activity questionnaire: 12-country reliability and validity. Med Sci Sports Exerc.

[34] Moghaddam MB (2012). The Iranian version of international physical activity questionnaire (IPAQ) in Iran: content and construct validity, factor structure, internal consistency and stability. World Appl Sci.

[35] Ainsworth BE (2000). Compendium of physical activities: an update of activity codes and MET intensities. Med Sci Sports Exerc.

[36] Esfahani FH (2010). Reproducibility and relative validity of food group intake in a food frequency questionnaire developed for the Tehran lipid and glucose study. J Epidemiol.

[37] Djazayery A, Pajooyan J (2000). Food consumption patterns and nutritional problems in the Islamic Republic of Iran. Nutr Health.

[38] Ghafarpour M, Houshiar-Rad A, Kianfar H (1999). The manual for household measures, cooking yields factors and edible portion of food.

[39] Mirmiran P (2010). Reliability and relative validity of an FFQ for nutrients in the Tehran lipid and glucose study. Public Health Nutr.

[40] Willett WC, Howe GR, Kushi LH (1997). Adjustment for total energy intake in epidemiologic studies. Am J Clin Nutr.

[41] Newby P, Tucker KL (2004). Empirically derived eating patterns using factor or cluster analysis: a review. Nutr Rev.

[42] Ongagna J (2001). The HLA-DQB alleles and amino acid variants of the vitamin D-binding protein in diabetic patients in Alsace. Clin Biochem.

[43] Borges CR (2010). Full-length characterization of proteins in human populations. Clin Chem.

[44] Lee DM (2009). Vitamin D, parathyroid hormone and the metabolic syndrome in middle-aged and older European men. Eur J Endocrinol.

[45] Hirai M (2000). Variations in vitamin D-binding protein (group-specific component protein) are associated with fasting plasma insulin levels in Japanese with normal glucose tolerance. J Clin Endocrinol Metab.

[46] Arnaud J, Constans J (1993). Affinity differences for vitamin D metabolites associated with the genetic isoforms of the human serum carrier protein (DBP). Hum Genet.

[47] Gozdzik A (2011). Association of vitamin D binding protein (VDBP) polymorphisms and serum 25(OH)D concentrations in a sample of young Canadian adults of different ancestry. J Steroid Biochem Mol Biol.

[48] Carpenter TO (2013). Vitamin D binding protein is a key determinant of 25-hydroxyvitamin D levels in infants and toddlers. J Bone Miner Res.

[49] Ahn J (2010). Genome-wide association study of circulating vitamin D levels. Hum Mol Genet.

[50] Wang TJ (2010). Common genetic determinants of vitamin D insufficiency: a genome-wide association study. Lancet.

[51] Baier LJ (1998). Variations in the vitamin D-binding protein (Gc locus) are associated with oral glucose tolerance in nondiabetic Pima Indians. J Clin Endocrinol Metab.

[52] Hirai M (1998). Group specific component protein genotype is associated with NIDDM in Japan. Diabetologia.

[53] Schneider A (2015). Vitamin D, vitamin D binding protein gene polymorphisms, race and risk of incident stroke: the atherosclerosis risk in communities (ARIC) study. Eur J Neurol.

[54] Phillips CM (2010). Gene-nutrient interactions with dietary fat modulate the association between genetic variation of the ACSL1 gene and metabolic syndrome. J Lipid Res.

[55] Streppel MT (2005). Dietary fiber and blood pressure: a meta-analysis of randomized placebo-controlled trials. Arch Intern Med.

[56] Whelton SP (2005). Effect of dietary fiber intake on blood pressure: a meta-analysis of randomized, controlled clinical trials. J Hypertens.

[57] van Dam RM (2002). Dietary patterns and risk for type 2 diabetes mellitus in US men. Ann Intern Med.

[58] Östman EM, Elmståhl HGL, Björck IM (2001). Inconsistency between glycemic and insulinemic responses to regular and fermented milk products. Am J Clin Nutr.

[59] Szathmary EJ (1987). The effect of Gc genotype on fasting insulin level in Dogrib Indians. Hum Genet.

[60] Lafi ZM (2015). Association of rs7041 and rs4588 polymorphisms of the vitamin D binding protein and the rs10741657 polymorphism of CYP2R1 with vitamin D status among jordanian patients. Genet Test Mol Biomark.

[61] Lauridsen AL (2005). Plasma concentrations of 25-hydroxy-vitamin D and 1,25-dihydroxy-vitamin D are related to the phenotype of Gc (vitamin D-binding protein): a cross-sectional study on 595 early postmenopausal women. Calcif Tissue Int.

[62] Sollid ST (2016). Effects of vitamin D binding protein phenotypes and vitamin D supplementation on serum total 25(OH)D and directly measured free 25(OH)D. Eur J Endocrinol.

[63] Taban IM (2017). Analysis of the binding sites of vitamin D 1α-hydroxylase (CYP27B1) and vitamin D 24-hydroxylase (CYP24A1) for the design of selective CYP24A1 inhibitors: homology modelling, molecular dynamics simulations and identification of key binding requirements. Bioorg Med Chem.

[64] Jolliffe DA (2016). Single nucleotide polymorphisms in the vitamin D pathway associating with circulating concentrations of vitamin D metabolites and non-skeletal health outcomes: review of genetic association studies. J Steroid Biochem Mol Biol.

[65] Tan C-E (2004). Can we apply the national cholesterol education program adult treatment panel definition of the metabolic syndrome to Asians?. Diabetes Care.

